# Smegma in diabetes mellitus

**DOI:** 10.11604/pamj.2021.40.94.26196

**Published:** 2021-10-13

**Authors:** Thamarai Kannan Sampath, Krishna Prasanth Baalan

**Affiliations:** 1 Department of Community Medicine, Sree Balaji Medical College and Hospital, Biher Chennai, India

**Keywords:** Sexually transmitted infection, diabetes mellitus, smegma

## Image in medicine

A 37-year-old male patient presented with 1-month history of pain over the bulb of penis during retraction of foreskin. Patient suffered from type 1 diabetes mellitus on poor glycemic control. On examination multiple white patches of 1 mm x 3 mm dimension were observed with pain during retraction of prepuce. Smegma deposition over the glans penis and erythematous areas were revealed while scraping the lesions. The patient, screened for urinary tract infection (UTI) and sexually transmitted disease (STD) including hepatitis B, syphilis and HIV which were negative and complete blood count was normal. Since smegma can be a precursor for genital infections, physicians must scrupulously examine diabetic patients presenting as timely diagnosis and treatment would improve patient´s quality of life. The patient, put on long acting insulin and advised personal hygiene and showed significant improvement during his follow-up visit, 1 month later.

**Figure 1:**
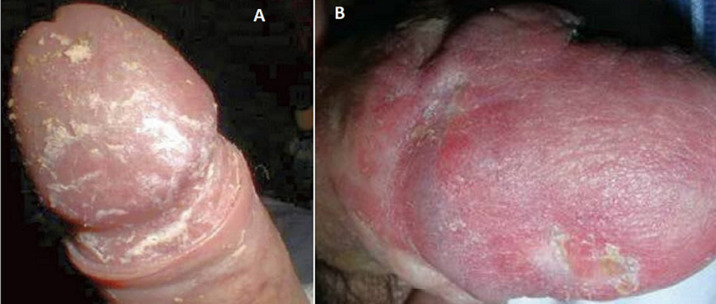
A) smegma deposition over the glans penis; B) erythematous lesions secondary to scratch were seen on the affected region

